# ESR Essentials: screening for breast cancer - general recommendations by EUSOBI

**DOI:** 10.1007/s00330-024-10740-5

**Published:** 2024-04-24

**Authors:** Magda Marcon, Michael H. Fuchsjäger, Paola Clauser, Ritse M. Mann

**Affiliations:** 1 Institute of Diagnostic and Interventional Radiology, University Hospital Zurich, Raemistrasse 100, 8091 Zurich, Switzerland; 2 Institute of Radiology, Hospital Lachen, Oberdorfstrasse 41, 8853 Lachen, Switzerland; 3 Division of General Radiology, Department of Radiology, Medical University Graz, Auenbruggerplatz 9, 8036 Graz, Austria; 4 Department of Biomedical Imaging and Image-guided Therapy, Division of General and Pediatric Radiology, Research Group: Molecular and Gender Imaging, Medical University of Vienna, Währinger Gürtel 18-20, 1090 Wien, Austria; 5 Department of Diagnostic Imaging, Radboud University Medical Centre, Geert Grotteplein Zuid 10, 6525 GA Nijmegen, The Netherlands

**Keywords:** Breast neoplasms, Mammography, Early detection of cancer, Ultrasonography (mammary), Magnetic resonance imaging

## Abstract

**Abstract:**

Breast cancer is the most frequently diagnosed cancer in women accounting for about 30% of all new cancer cases and the incidence is constantly increasing. Implementation of mammographic screening has contributed to a reduction in breast cancer mortality of at least 20% over the last 30 years. Screening programs usually include all women irrespective of their risk of developing breast cancer and with age being the only determining factor. This approach has some recognized limitations, including underdiagnosis, false positive cases, and overdiagnosis. Indeed, breast cancer remains a major cause of cancer-related deaths in women undergoing cancer screening. Supplemental imaging modalities, including digital breast tomosynthesis, ultrasound, breast MRI, and, more recently, contrast-enhanced mammography, are available and have already shown potential to further increase the diagnostic performances. Use of breast MRI is recommended in high-risk women and women with extremely dense breasts. Artificial intelligence has also shown promising results to support risk categorization and interval cancer reduction. The implementation of a risk-stratified approach instead of a “one-size-fits-all” approach may help to improve the benefit-to-harm ratio as well as the cost-effectiveness of breast cancer screening.

**Key Points:**

- *Regular mammography should still be considered the mainstay of the breast cancer screening.*
- *High-risk women and women with extremely dense breast tissue should use MRI for supplemental screening or US if MRI is not available.*
- *Women need to participate actively in the decision to undergo personalized screening.*

**Key recommendations:**

- Mammography is an effective imaging tool to diagnose breast cancer in an early stage and to reduce breast cancer mortality (evidence level I). Until more evidence is available to move to a personalized approach, regular mammography should be considered the mainstay of the breast cancer screening.
- High-risk women should start screening earlier; first with yearly breast MRI which can be supplemented by yearly or biennial mammography starting at 35–40 years old (evidence level I). Breast MRI screening should be also offered to women with extremely dense breasts (evidence level I). If MRI is not available, ultrasound can be performed as an alternative, although the added value of supplemental ultrasound regarding cancer detection remains limited.
- Individual screening recommendations should be made through a shared decision-making process between women and physicians.

## Introduction

More than 2 million women are diagnosed with breast cancer worldwide every year, and the estimated 5-year prevalence is almost 8 million cases. In Europe, breast cancer is the most frequently occurring cancer, accounting for about 15% of all new cancer cases and for about 30% of all new cancer cases in women. More than five hundred thousand breast cancers are diagnosed every year in Europe, and about 13% of women will be diagnosed with breast cancer during their lifetime. Breast cancer more frequently affects women older than 50 years [1]. Nevertheless, the global incidence has constantly increased in the last years in all age groups, with some regional and age group differences [2]. Although not completely clear, this trend could be explained by the introduction of organized screening programs as well as modification in exposure to risk factors, such as hormonal and reproductive factors or smoking. Improvements in diagnosis and treatment have significantly reduced breast cancer mortality in the last three decades. Nevertheless, breast cancer mortality remains an important issue, and about 3% of women will eventually die from breast cancer; the estimated number of deaths for breast cancer in Europe in 2020 was about 140,000 women [3].

### Risk stratification

Multiple factors influence the individual risk to develop breast cancer, including age at menarche and menopause, reproductive history, obesity, previous biopsy with atypia, previous thoracic radiation therapy, and family cancer history [4]. Breast tissue composition is also a determining factor. Women with higher mammographic breast density, defined as the proportion of radiopaque fibroglandular tissue compared to radiolucent adipose tissue in the breast, have a 2.9-6-fold increased risk of developing breast cancer compared to women with predominantly fatty breast [5]. Several risk prediction models are available to estimate the individual risk of developing breast cancer [6–8]. These models take into account a number of personal factors and can include genetic information, such as expression of high- (e.g., BRCA1, BRCA2, TP53) and low-penetrance (e.g., single-nucleotide polymorphisms, SNPs) genes, as well as the mammographic breast density [7, 9]. Recent studies have shown that the integration of deep learning models applied to mammographic images can further improve risk assessment [10].

Women with a lifetime risk of developing breast cancer of less than 15% are considered at average risk [11]. Women with a previous biopsy showing atypical ductal hyperplasia or lobular carcinoma in situ, as well as women with a previous history of breast cancer, are included in the intermediate risk category with an estimated lifetime risk of 15–20%. The high-risk category includes all women with a lifetime risk to develop breast cancer of more than 20%. However, this is usually split into an intermediate high and a very high category. The intermediate high category includes women with a highly positive family history but no known mutations, as well as those with lower penetrance genetic mutations such as CHEK2 or BARD1. Very high risk—with a lifetime risk of over 50%—is usually due to hereditary mutations in high penetrance genes and is also present in women who underwent chest radiation therapy between the ages of 10–30 years. A genetic predisposition is observed in about 5–10% of breast cancers, and BRCA1 and BRCA2 are the most recognized mutations associated with a lifetime risk for breast cancer of 50–85% and 45–69%, respectively [12]. Dedicated screening recommendations are available for high-risk women, but sometimes differ between women at very high risk and those with an intermediate high risk [13]. Since women at very high risk should start screening from the age of 25, early risk assessment is important. Ongoing clinical trials, e.g., the MyPeBS (My Personal Breast Cancer Screening) trial in Europe and the WISDOM (Women Informed to Screen Depending on Measures of Risk) trial in the United States, are investigating the implementation of different screening modalities and schedules based on personal breast cancer risk estimation for women that are not known to be at a very high risk [14, 15].

### The idea behind screening: downsizing and downstaging

The goal of breast cancer screening is to find relevant cancers when they are so small that they do not cause symptoms and are less likely to have spread beyond the location where they originated [16]. In the past, breast self-examination has been recommended for cancer prevention as an inexpensive and noninvasive mean to perform routine screening. Nevertheless, studies have shown that its regular practice is not effective at reducing breast cancer mortality [16, 17]. Mammography meets the characteristics of a screening test: it can be rapidly and easily applied, it is broadly available, and it was shown that the test allows for early detection of breast cancers in several randomized controlled clinical trials that ultimately also demonstrated an estimated breast cancer-related mortality reduction of at least 20–25% [18]. As such, these studies show that downstaging of breast cancer through screening improves survival. However, many factors are involved to determine breast cancer prognosis, including cancer biological features (e.g., tumor subtype and grade, hormone receptor, and human epidermal growth factor receptor 2 status) and the availability of effective therapies. Indeed, the parallel spread of screening programs, the evolution of breast surgical approaches, and the implementation of more effective systemic treatments make the determination of the relative contribution of each factor on the achieved mortality reduction more challenging. Regardless, treatments remain less effective in the case of advanced-stage disease, and mortality reduction is still significantly related to the tumor size and nodal status at diagnosis, which supports screening for breast cancer to pursue an early diagnosis [19, 20].

To assess whether alternative screening methods are effective, pursuing randomized controlled trials with mortality as the endpoint is nowadays regarded as impossible and unethical. Due to the earlier detection on the one hand and the long-lasting effects of palliative therapies in women with (incurable) metastatic disease, on the other hand, the timespan between diagnosis and eventual death is too long to assess the ongoing rapid technological developments in screening methodology. Therefore, surrogate endpoints need to be evaluated that are prognostic for the eventual survival. These include the observation of: 1) a stage shift (i.e., cancers detected through a new screening modality are smaller and less often node positive than cancers detected by the standard modality or through symptoms); 2) a reduction in the frequency of interval cancers (i.e., a decrease in the fraction of cancers that present through symptoms between two screening rounds); and 3) a reduction in the relative frequency of advanced cancers (classically regarded as a reduction in stage III and IV cancers). The achieved mortality reduction and associated cost-effectiveness of screening can subsequently be modeled from either of these outcomes.

### Screening methods

#### Mammography

**Fig. 1:**
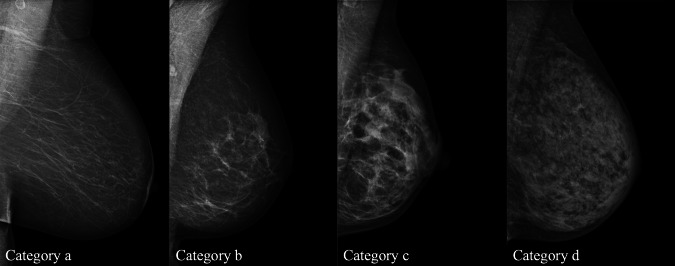
Breast density categories according to the Breast Imaging Reporting and Data System (BI-RADS) from the American College of Radiology [57]. Depending on the breast composition, four different categories are identified: **a** entirely fatty; **b** scattered areas of fibroglandular density; **c** heterogeneously dense, which may obscure masses; and (**d**) extremely dense

**Fig. 2:**
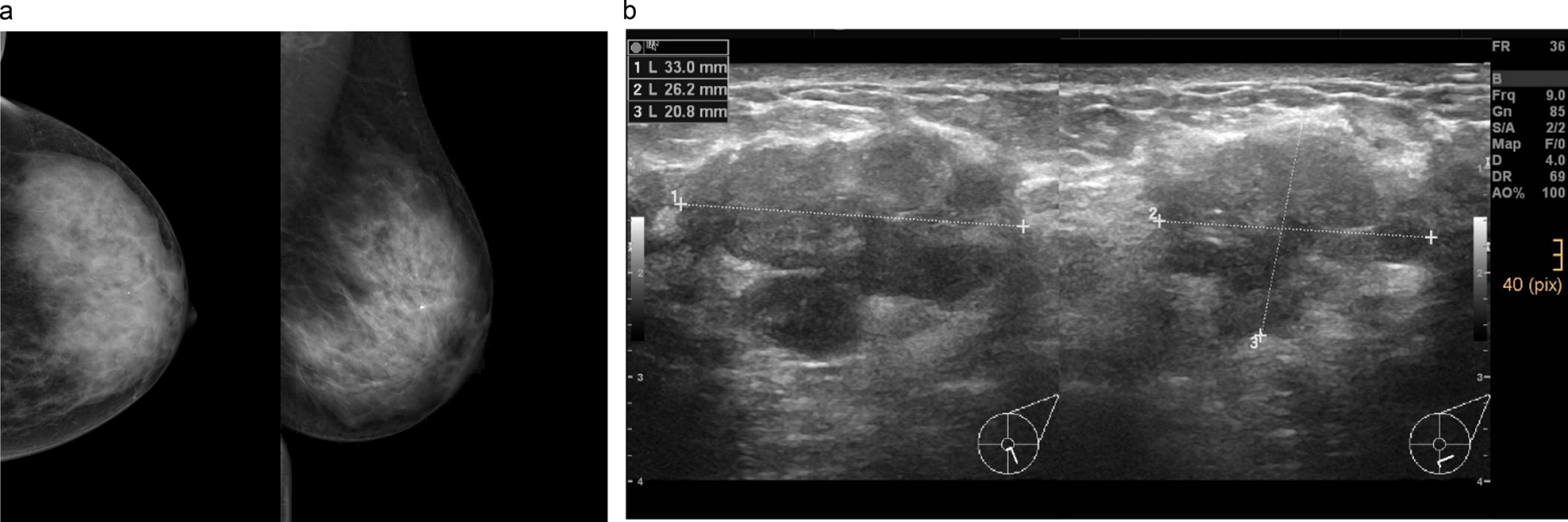
45-year-old woman without significant family/personal risk factors for breast cancer undergoing first breast cancer screening examination. In the left mammogram (**a** cranio-caudal and mediolateral oblique projections), extremely dense breast tissue can be observed, and no suspicious findings could be identified, only a typical benign calcification can be recognized in the retromamillary region. At supplemental ultrasound (**b**) performed on the same day, a suspicious mass up to 3.3 cm could be identified at 6 o’clock in the left breast. Ultrasound-guided biopsy was performed, and the lesion histologically corresponded to a NST moderately differentiated invasive ductal carcinoma

**Fig. 3:**
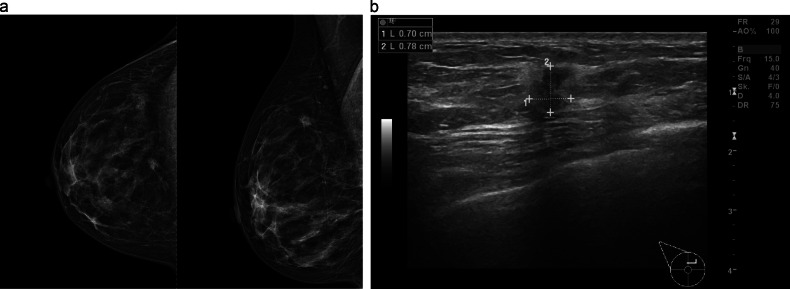
55-year-old woman without significant family/personal risk factors for breast cancer undergoing routine breast cancer screening examination. In the right mammogram (**a** cranio-caudal and mediolateral oblique projections) scattered areas of fibroglandular density can be observed. A suspicious irregular shaped and spiculated mass can be seen at 12 o’clock. At ultrasound a corresponding irregular shaped, spiculated and hypoechoic mass up to 0.8 cm can be seen (**b**). Ultrasound-guided biopsy was performed and the mass histologically corresponded to a NST moderately differentiated invasive ductal carcinoma

The most common method of screening women for breast cancer is mammography, either offered by an organized program or in the form of opportunistic screening. In most European countries, mammographic screening programs include all women, with age being the only determinant: screening is offered starting at the age of 40–50 years until the age of 64–74 years or even illimitable if women are in good health (e.g., in Austria) at intervals of 1–2 years (3 years in the UK). Population-based effectiveness of mammography screening programs depend on high participation levels, and the European Guidelines for Quality Assurance in Breast Cancer Screening and Diagnosis recommend that at least 70–75% of a population participates in regular mammography screening [21]. Despite the overall survival advantages achieved with the use of mammographic screening, this “one-size-fits-all” approach is associated with several drawbacks, not least that mammography alone is not sufficient to achieve early diagnosis in the high-risk population. It has been reported that program sensitivities of 86–89% in women with largely fatty breasts drop to 62–68% in the case of women with dense breasts (Fig. 1–3) [22]. Considering that about 40–50% of women have dense breasts (heterogeneously and extremely dense breast), this represents a non-negligible issue. Breast density category after performing mammography screening should always be reported since this has important implications for the performance of supplemental and/or alternative imaging methods.

#### Digital breast tomosynthesis

Digital breast tomosynthesis (DBT) is a pseudo-3D X-ray-based imaging technique that involves multiple low-dose projections acquired across an arc over the breast that are subsequently reconstructed into a series of stacked images, as well as a synthetic mammogram. The masking effect is reduced by decreasing superimposition from overlying breast tissue, thus increasing cancer detection rates (up to 4 additional cancers detected per 1000 screens) compared to mammography. Decreased recall rates compared to mammography have also been reported in high recall settings (i.e., most studies in the United States), whereas no differences or even slightly higher recall rates with DBT have been reported in most European studies [23, 24]. Based on these results, DBT is increasingly used for breast cancer screening in Europe, as well as in the United States. Nevertheless, several studies have shown that the positive effects are limited in women with very high breast density and also that the reported increased detection of slow-growing, probably indolent tumors generate concerns related to overdiagnosis (see below). Moreover, the studies investigating the role of DBT on the interval cancer rate still show mixed results [25–28]. When interpreting DBT images, a 2D image of the same projection is required e.g. for comparison with prior mammogram and assessment of calcifications. For this purpose, mammography projections were often  acquired at the same time. Studies have demonstrated that synthetic mammography, corresponding to 2D reconstructions of the DBT datasets, can be used as an alternative to additional mammography projection without impairing screening performances and reducing radiation exposure as well as image acquisition time [29].

#### Breast ultrasound

Several studies have shown that supplemental breast ultrasound screening in women with dense breasts increases the detection of 0.9–7.7 cancers per 1000 screening examinations and reduces the interval cancer rate compared to mammography alone. These detected cancers are usually small, invasive, and node-negative. In the last decade, automated breast ultrasound systems have also been introduced on the market to overcome some of the limitations of handheld ultrasound, such as operator dependence, long acquisition time when performed by a physician, and limited reproducibility. The main drawbacks of supplemental breast ultrasound screening remain the increase in recalls and biopsy rates, leading to a reduction of the positive predictive value of screening to about 5% [30]. Mostly due to the long examination times, the cost-effectiveness of ultrasound for screening is questionable. EUSOBI (European Society of Breast Imaging) guidelines suggest the possible usage of supplemental ultrasound screening in women at average or intermediate risk with dense breast and negative mammography, but the balance between advantages and disadvantages was not judged to be clear enough to adopt additional ultrasound as a general policy [31].

#### MRI

**Fig. 4:**
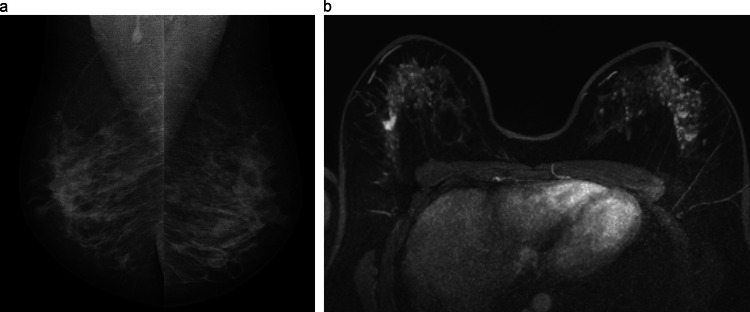
46-year-old patient with a prior history of chest irradiation for Ewing sarcoma. Screening examinations. MLO views (**a**) from mammography examination that was deeemed normal. Contrast-enhanced breast MRI (**b**) reveals bilateral breast abnormalities. In the right breast, an irregular spiculated mass is visible, corresponding to a 13 mm NST carcinoma. In the left breast, segmental heterogeneous nonmass enhancement is seen, corresponding to extensive DCIS with microinvasion

Breast MRI is the most sensitive imaging method for breast cancer detection, mostly because of its ability to detect neovascularity. Moreover, as a functional imaging modality, it is optimized to detect more aggressive tumor subtypes [32–35]. In addition, the sensitivity of breast MRI is not limited by breast density. Screening with breast MRI is recommended yearly for women with genetics-based increased risk, with a calculated lifetime risk equal or superior to 20% and in case of a history of chest or mantle radiation therapy (Fig. 4). From the age of 35–40 years, supplemental mammography may also be considered. Recently, based on the Dutch DENSE trial and the international EA1411 ECOG-ACRIN study, the EUSOBI has published the recommendations for breast cancer screening in women with extremely dense breast (category D according to the ACR BI-RADS atlas) [36–40]. For this group of women, level I evidence is now available that MRI screening cost-effectively reduces mortality. Based on this evidence, healthcare systems should focus on building programs that can offer MRI from age 50 to 70, at least every 4 years, in women with very dense breasts. Major drawbacks of breast MRI remain higher costs of the screening program, relatively limited availability that currently requires expansion, and long examination time, although the latter seems to be solved by abbreviated protocols that are equally effective and enable imaging in a similar timespan as mammography.

#### Contrast-enhanced mammography

Contrast-enhanced Mammography (CEM) is a recently introduced breast imaging technique that like breast MRI uses contrast medium with the purpose to depict vascularity potentially associated with breast cancer, thus providing anatomic and functional information at the same time. In the screening setting, few studies so far have investigated the use of CEM, particularly among women with intermediate/high lifetime risk [41, 42]. CEM shows superior diagnostic performance compared with digital mammography, DBT, and mammography with supplemental ultrasound and seems to show equal to a bit higher specificity [16] but lower sensitivity compared with breast MRI. Thus, CEM seems a promising alternative to breast MRI when MRI is not available. Nevertheless, most studies published so far are retrospective and/or include a limited number of women. Further investigations on a larger scale are necessary to compare CEM with standard imaging, and particularly MRI, in terms of achieved stage shift and frequency of interval and advanced cancers [43].

**Fig. 5:**
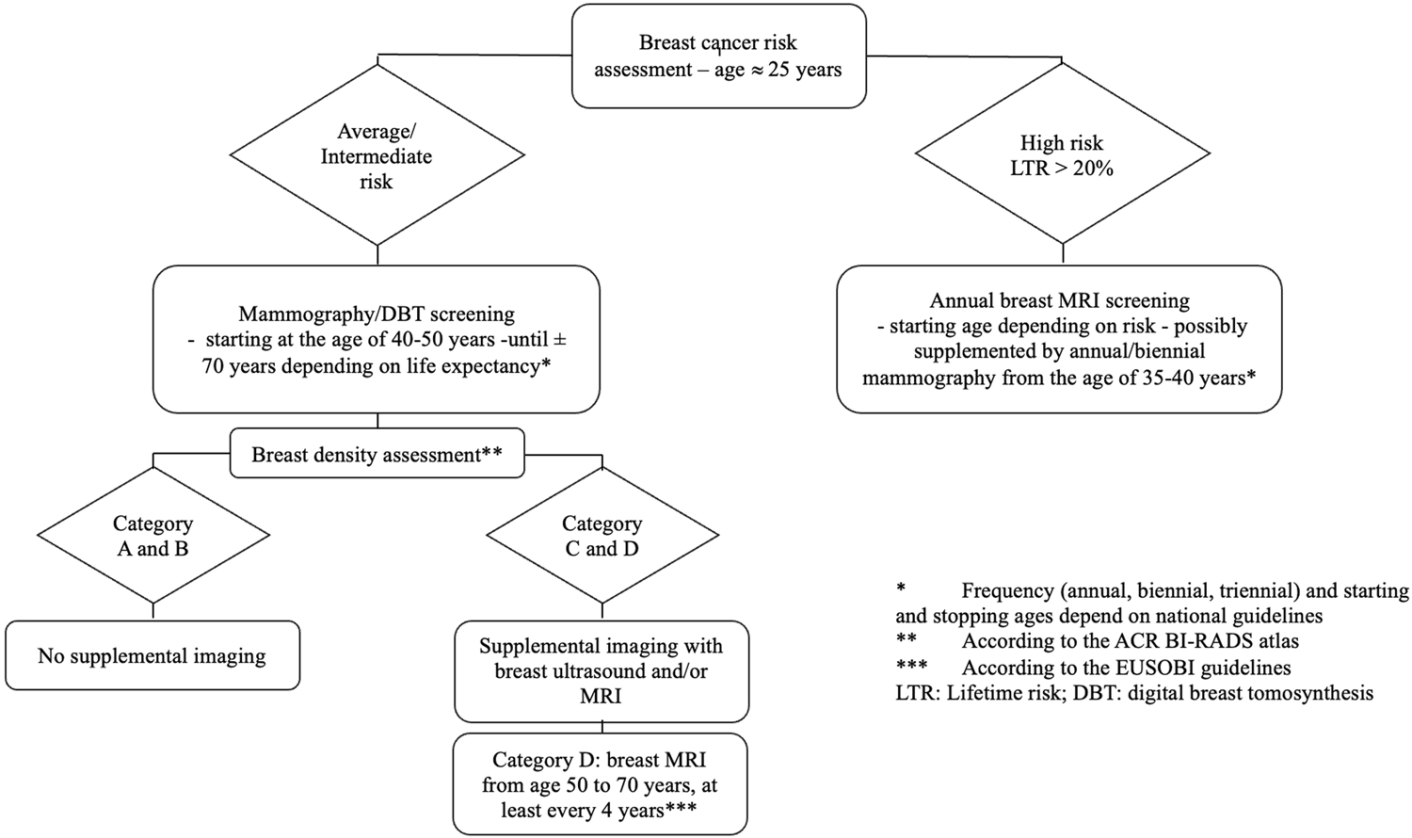
Flowchart summarizing the clinical pathways for breast cancer screening in different women subgroups

Figure 5 summarizes the suggested clinical pathways for breast cancer screening in different women subgroups.

### The downsides of screening

Despite the evidence that mammography screening programs reduce mortality from breast cancer, the net benefit is counterbalanced by some recognized factors, namely underdiagnosis, false positive cases, and overdiagnosis.

#### Underdiagnosis

Underdiagnosis is the situation where a clinically relevant breast cancer is present in the breast but is not detected at the time of screening. This leads to cancer detection at a later time when the cancer has progressed and is less easily—and sometimes also less successfully—treatable. Classically, interval cancers are counted as a false negative or underdiagnosed cases. This accounts for approximately 15–40% of cancers detected within a population screened with mammography, mostly depending on breast density. However, underdiagnosis is more frequent as approximately 30% of cancers detected through mammography screening already have spread to the lymph nodes, and 25% are over 2 cm in size (i.e., T2 or more). Retrospective evaluation of screening mammograms shows that 30–50% of these cancers were already visible on the preceding mammogram, but not recognized at that time. Studies with other screening modalities, particularly breast MRI, also show that some cancers could be found earlier when they are smaller and lymph node-negative. Whether this has implications for treatment and prognosis depends also on the biological profile of the cancer. Unfortunately, thus far, it is not possible to predict what type of cancer a woman will develop, and consequently, the only remedy against underdiagnosis is to try to find all cancers as early as possible [8, 44].

#### False positive

Up to 10–12% of women undergoing screening mammography are recalled for additional workup, which may consist of additional imaging, tissue biopsy, or short-term follow-up. Among these recalls, only about one-third of cases will have a breast cancer diagnosis, whereas the others may have a benign lesion or a finding caused by tissue superimposition. These are referred to as false positive cases [8, 44]. The probability of a false positive result is higher in the case of a baseline mammogram. [45]. The European quality assurance guidelines for breast cancer screening recommend double reading, with both readers initially performing independent mammogram evaluation, followed by resolving discordant results by third reader arbitration or consensus between the two readers [46]. Compared to single reading, double reading reduces the recall rate without reducing the cancer detection rate [47]. False positive cases are considered a harm of screening because of medical costs and discomfort experienced by the patient. Moreover, the rate of participation to future screening rounds may decrease after experiencing a false positive result [48]. To reduce anxiety to a minimum, it is of paramount importance to quickly analyze recalled women.

#### Overdiagnosis

Overdiagnosis refers to the detection of breast cancers that would never become clinically evident and would not cause any harm during the individual’s lifetime if left untreated. It is, unfortunately, never possible to directly tell which cancers have been “overdiagnosed”. Slower-growing and lower-grade cancers have a higher probability of being overdiagnosed, causing a length bias. Moreover, these cancers are more often associated with microcalcifications and with a stromal reaction (spiculation), which make their identification in mammography easier [36], and, which makes mammography screening prone to overdiagnosis. Overdiagnosis leads to overtreatment and possibly causes morbidity or even mortality without any certain benefit. It is difficult to estimate the extent of overdiagnosis related to mammography screening, as multiple factors are involved, such as age, cancer risk of the screened population, and lead time. Previous published works reported percentages of overestimation varying between 0 and 50% of all detected cancer: this variation also depended on different definitions of overdiagnosis, study settings, and estimation methods [36]. In a more recent study, accounting for the detection of nonprogressive cancer, about 15% of screen-detected cancers were estimated to be overdiagnosed [36]. The impact of overdiagnosis may be reduced by identifying women with “low-risk disease” who could undergo active monitoring instead of surgery or even who do not necessitate further investigations at the point of screening, thus reducing overtreatment. Currently, several ongoing trials are evaluating the use of active monitoring instead of conventional surgical treatment in women with a diagnosis of low-risk ductal carcinoma in situ [49–51].

### Economic considerations

Although the cost-effectiveness of breast cancer screening is dependent on local costs of imaging techniques and labor as well as the value attributed to increased life expectancy and overall health, economic models suggest that breast cancer screening in the European healthcare setting improves life and/or quality-adjusted life expectancy at acceptable costs. Risk-adapted screening strategies should guarantee further optimization of the resource utilization by assigning each risk category the most advantageous and cost-effective screening method, thereby reducing unnecessary harm and costs [52, 53].

In countries with limited resource availability, screening tools need to be chosen wisely depending on the cost-effectiveness and performance expected in the population. In situations where financial constraints limit the implementation of large-scale screening, priority should be given to raising breast cancer awareness and ensuring timely presentation of breast complaints to radiologists, potentially by education on physical breast examination and modifiable breast cancer risk factors [54].

### Women preferences

Women should be properly informed about the advantages as well as disadvantages of a screening test to be able to make informed choices. To minimize anxiety, prompt provision of screening results is also important [55]. Shared decision-making is characterized by the participation of physicians and patients on taking the best decision, based not only on the scientific evidence but also on expectations and wishes of each patient. Breast radiologists should embrace an active role in this, as knowledge about the benefits and harms of different screening scenarios is not common among other physicians. Several studies on population-based mammography screening have shown that women value more the possibility of early diagnosis over the risk of overdiagnosis or a false positive exam, although concerns remain because the meaning of overdiagnosis may not be fully understood [56]. Moving to the scenario of risk-based cancer screening, potentially encompassing a more intensive screening for women at higher risk and less intensive screening for women at lower risk, informed decision-making could become even more important to increase acceptability among those women who are candidates for reduced screening intensity [57].

### General recommendations

Screening mammography is an effective tool to reduce breast cancer mortality (evidence level I). Existing screening programs should be continued, and efforts should be made to increase adherence. Digital breast tomosynthesis can be performed as an alternative to mammography.

High-risk women should start screening as early as 25 years of age with yearly breast MRI (evidence level I), supplemented with mammography from age 35–40 years.

Women at intermediate risk of breast cancer may benefit from supplemental screening, including digital breast tomosynthesis, breast ultrasound, breast MRI, and possibly CEM. The most appropriate imaging modalities should be adjusted to patient characteristics. The current evidence supports the use of breast MRI screening in women with extremely dense breast tissue, preferably every 2–3 years (evidence level I). If MRI is not available, supplemental ultrasound can be performed as an alternative, although the added value of supplemental ultrasound regarding cancer detection remains more limited.

Several risk prediction models are available to estimate the individual risk of developing breast cancer. If possible, risk assessment should be performed at a young age (approximately 25 years) to effectively tailor screening recommendations. Individual screening recommendations should be made through a shared decision-making process between women and physicians.

### Summary statement

Tumor stage at diagnosis remains a crucial prognostic factor to determine breast cancer survival. By means of mammographic screening, early cancer diagnosis can be achieved thereby reducing breast cancer mortality and the need for aggressive treatments. Nevertheless, breast cancer remains the leading cause of cancer death among women worldwide, and although the benefits of mammographic screening outweigh the harms in the general population and the “one-size-fit-all” approach remains easier to implement, further improvements are sought.

Moving to screening programs adjusted to personal risk level instead of age-based population screening could improve the performance of a screening program by reducing underdiagnosis, false positives, and overdiagnosis as well as improving cost-effectiveness.

Other effective imaging modalities are available and, depending on the characteristics of the screened population, could be implemented as an alternative to mammography (e.g., breast tomosynthesis and breast MRI) or as a supplemental tool (e.g., breast ultrasound) (Table 1). Although these additional examinations may be susceptible to higher false positive rates compared to mammography, considering the potentially dramatical consequences of a late cancer diagnosis, priority should be given to avoid false negative examinations rather than to reduce false positive.

**Table 1:** Summary recommendations on breast cancer screening

Recommendations on breast cancer screening
• Regular mammography should be considered the mainstay of breast cancer screening (evidence level I); digital breast tomosynthesis can be performed as an alternative.
• Women at high risk of breast cancer: screening should start as early as 25 years of age with annual breast MRI (evidence level I), supplemented with mammography from age 35 to 40 years.
• Women at intermediate risk of breast cancer: supplemental screening, including digital breast tomosynthesis, breast ultrasound, breast MRI, and possibly contrast-enhanced mammography may be beneficial. The most appropriate imaging modalities should be adjusted to patient characteristics.
• Women with extremely dense breast tissue: supplemental screening with MRI should be performed preferably every 2–3 years (evidence level I). If MRI is not available, supplemental ultrasound can be performed as an alternative although the evidence remains more limited.
• Whenever possible, risk assessment should be performed at a young age (≈25 years) to effectively tailor screening recommendations.

### Patient summary

Breast cancer screening can detect small cancers before they become symptomatic, leading to, in most cases, less aggressive treatment and prolonged survival. So far, breast cancer screening has mainly been performed through mammography, which still represents a very effective imaging modality but has recognized weaknesses. For example, the performance of a screening exam can vary depending on personal characteristics such as the family history and the breast density. There is now evidence that considering these and some other factors to provide a more personalized screening and incorporating other screening modalities may help to further improve the early diagnosis of breast cancer. Several current studies are investigating these aspects.

## Abbreviations

DENSE trial: The Dense Tissue and Early Breast Neoplasm Screening
EUSOBI: European Society of Breast Imaging
MyPeBS trial: My Personal Breast Cancer Screening trial
WISDOM trial: Women Informed to Screen Depending on Measures of Risk

## References

[1] IARC Cancer Today. International Agency for Research on Cancer Centers for disease Control and prevention Accessed September 7, 2023

[2] Lima SM, Kehm RD, Terry MB (2021) Global breast cancer incidence and mortality trends by region, age-groups, and fertility patterns. EClinicalMedicine 38:100985 34278281 10.1016/j.eclinm.2021.100985 PMC8271114

[3] Dyba T, Randi G, Bray F et al (2021) The European cancer burden in 2020: Incidence and mortality estimates for 40 countries and 25 major cancers. Eur J Cancer 157:308–347 34560371 10.1016/j.ejca.2021.07.039 PMC8568058

[4] Lukasiewicz S, Czeczelewski M, Forma A, Baj J, Sitarz R, Stanislawek A (2021) Breast cancer-epidemiology, risk factors, classification, prognostic markers, and current treatment strategies-an updated review. Cancers (Basel) 13:4287 10.3390/cancers13174287 PMC8428369 34503097

[5] McCormack VA, dos Santos Silva I (2006) Breast density and parenchymal patterns as markers of breast cancer risk: a meta-analysis. Cancer Epidemiol Biomarkers Prev 15:1159–1169 16775176 10.1158/1055-9965.EPI-06-0034

[6] Gail MH, Brinton LA, Byar DP et al (1989) Projecting individualized probabilities of developing breast cancer for white females who are being examined annually. J Natl Cancer Inst 81:1879–1886 2593165 10.1093/jnci/81.24.1879

[7] Tyrer J, Duffy SW, Cuzick J (2004) A breast cancer prediction model incorporating familial and personal risk factors. Stat Med 23:1111–1130 15057881 10.1002/sim.1668

[8] Lee CS, Bhargavan-Chatfield M, Burnside ES, Nagy P, Sickles EA (2016) The National Mammography Database: preliminary data. AJR Am J Roentgenol 206:883–890 26866649 10.2214/AJR.15.14312

[9] Lee A, Mavaddat N, Wilcox AN et al (2019) BOADICEA: a comprehensive breast cancer risk prediction model incorporating genetic and nongenetic risk factors. Genet Med 21:1708–1718 30643217 10.1038/s41436-018-0406-9 PMC6687499

[10] Yala A, Lehman C, Schuster T, Portnoi T, Barzilay R (2019) A deep learning mammography-based model for improved breast cancer risk prediction. Radiology 292:60–66 31063083 10.1148/radiol.2019182716

[11] Allweis TM, Hermann N, Berenstein-Molho R, Guindy M (2021) Personalized screening for breast cancer: rationale, present practices, and future directions. Ann Surg Oncol 28:4306–4317 33398646 10.1245/s10434-020-09426-1

[12] Ford D, Easton DF, Stratton M et al (1998) Genetic heterogeneity and penetrance analysis of the BRCA1 and BRCA2 genes in breast cancer families. The Breast Cancer Linkage Consortium. Am J Hum Genet 62:676–689 9497246 10.1086/301749 PMC1376944

[13] Monticciolo DL, Newell MS, Moy L, Lee CS, Destounis SV (2023) Breast cancer screening for women at higher-than-average risk: updated recommendations from the ACR. J Am Coll Radiol 20:902–914 37150275 10.1016/j.jacr.2023.04.002

[14] Roux A, Cholerton R, Sicsic J et al (2022) Study protocol comparing the ethical, psychological and socio-economic impact of personalised breast cancer screening to that of standard screening in the “My Personal Breast Screening” (MyPeBS) randomised clinical trial. BMC Cancer 22:507 35524202 10.1186/s12885-022-09484-6 PMC9073478

[15] Esserman L, Eklund M, Veer LV et al (2021) The WISDOM study: a new approach to screening can and should be tested. Breast Cancer Res Treat 189:593–598 34529196 10.1007/s10549-021-06346-w

[16] Organ) WWH (Accessed September 13, 2023) https://www.who.int/europe/news-room/fact-sheets/item/cancer-screening-and-early-detection-of-cancer

[17] Hackshaw AK, Paul EA (2003) Breast self-examination and death from breast cancer: a meta-analysis. Br J Cancer 88:1047–1053 12671703 10.1038/sj.bjc.6600847 PMC2376382

[18] Marmot MG, Altman DG, Cameron DA, Dewar JA, Thompson SG, Wilcox M (2013) The benefits and harms of breast cancer screening: an independent review. Br J Cancer 108:2205–2240 23744281 10.1038/bjc.2013.177 PMC3693450

[19] Smith RA, Duffy SW, Gabe R, Tabar L, Yen AM, Chen TH (2004) The randomized trials of breast cancer screening: what have we learned? Radiol Clin North Am 42:793–806 15337416 10.1016/j.rcl.2004.06.014

[20] Trimboli RM, Giorgi Rossi P, Battisti NML et al (2020) Do we still need breast cancer screening in the era of targeted therapies and precision medicine? Insights Imaging 11:105 32975658 10.1186/s13244-020-00905-3 PMC7519022

[21] Union POotE (2017) European guidelines for breast cancer screening and diagnosis: the European breast guidelines. Available via http://op.europa.eu/en/publication-detail/-/publication/b7b66c78-e139-11e6-ad7c-01aa75ed71a1/language-en/format-PDF

[22] Freer PE (2015) Mammographic breast density: impact on breast cancer risk and implications for screening. Radiographics 35:302–315 25763718 10.1148/rg.352140106

[23] Johnson K, Olinder J, Rosso A, Andersson I, Lang K, Zackrisson S (2023) False-positive recalls in the prospective Malmo Breast Tomosynthesis Screening Trial. Eur Radiol 33:8089–8099 37145147 10.1007/s00330-023-09705-x PMC10597871

[24] Marinovich ML, Hunter KE, Macaskill P, Houssami N (2018) Breast cancer screening using tomosynthesis or mammography: a meta-analysis of cancer detection and recall. J Natl Cancer Inst 110:942–949 30107542 10.1093/jnci/djy121

[25] Kerlikowske K, Su YR, Sprague BL et al (2022) Association of screening with digital breast tomosynthesis vs digital mammography with risk of interval invasive and advanced breast cancer. JAMA 327:2220–2230 35699706 10.1001/jama.2022.7672 PMC9198754

[26] Pattacini P, Nitrosi A, Giorgi Rossi P et al (2022) A randomized trial comparing breast cancer incidence and interval cancers after tomosynthesis plus mammography versus mammography alone. Radiology 303:256–266 35103537 10.1148/radiol.211132

[27] Johnson K, Lang K, Ikeda DM, Akesson A, Andersson I, Zackrisson S (2021) Interval breast cancer rates and tumor characteristics in the prospective population-based malmo breast tomosynthesis screening trial. Radiology 299:559–567 33825509 10.1148/radiol.2021204106

[28] Houssami N, Bernardi D, Caumo F et al (2018) Interval breast cancers in the ‘screening with tomosynthesis or standard mammography’ (STORM) population-based trial. Breast 38:150–153 29328943 10.1016/j.breast.2018.01.002

[29] Chikarmane SA, Offit LR, Giess CS (2023) Synthetic mammography: benefits, drawbacks, and pitfalls. Radiographics 43:e230018 37768863 10.1148/rg.230018

[30] van Zelst JCM, Mann RM (2018) Automated three-dimensional breast US for screening: technique, artifacts, and lesion characterization. Radiographics 38:663–683 29624482 10.1148/rg.2018170162

[31] Evans A, Trimboli RM, Athanasiou A et al (2018) Breast ultrasound: recommendations for information to women and referring physicians by the European Society of Breast Imaging. Insights Imaging 9:449–461 30094592 10.1007/s13244-018-0636-z PMC6108964

[32] Berg WA, Zhang Z, Lehrer D et al (2012) Detection of breast cancer with addition of annual screening ultrasound or a single screening MRI to mammography in women with elevated breast cancer risk. JAMA 307:1394–1404 22474203 10.1001/jama.2012.388 PMC3891886

[33] Sardanelli F, Boetes C, Borisch B et al (2010) Magnetic resonance imaging of the breast: recommendations from the EUSOMA working group. Eur J Cancer 46:1296–1316 20304629 10.1016/j.ejca.2010.02.015

[34] Kuhl CK, Schrading S, Bieling HB et al (2007) MRI for diagnosis of pure ductal carcinoma in situ: a prospective observational study. Lancet 370:485–492 17693177 10.1016/S0140-6736(07)61232-X

[35] Mann RM, Kuhl CK, Moy L (2019) Contrast-enhanced MRI for breast cancer screening. J Magn Reson Imaging 50:377–390 30659696 10.1002/jmri.26654 PMC6767440

[36] Mann RM, Athanasiou A, Baltzer PAT et al (2022) Breast cancer screening in women with extremely dense breasts recommendations of the European Society of Breast Imaging (EUSOBI). Eur Radiol 32:4036–4045 35258677 10.1007/s00330-022-08617-6 PMC9122856

[37] Veenhuizen SGA, de Lange SV, Bakker MF et al (2021) Supplemental breast MRI for women with extremely dense breasts: results of the second screening round of the DENSE trial. Radiology 299:278–286 33724062 10.1148/radiol.2021203633

[38] Comstock CE, Gatsonis C, Newstead GM et al (2020) Comparison of abbreviated breast MRI vs digital breast tomosynthesis for breast cancer detection among women with dense breasts undergoing screening. JAMA 323:746–756 32096852 10.1001/jama.2020.0572 PMC7276668

[39] Bakker MF, de Lange SV, Pijnappel RM et al (2019) Supplemental MRI screening for women with extremely dense breast tissue. N Engl J Med 381:2091–2102 31774954 10.1056/NEJMoa1903986

[40] D’Orsi, CJ, Sickles, EA, Mendelson, EB et al (2013) ACR BI-RADS® Atlas, Breast Imaging Reporting and Data System. Reston, VA, American College of Radiology

[41] Sorin V, Yagil Y, Yosepovich A et al (2018) Contrast-enhanced spectral mammography in women with intermediate breast cancer risk and dense breasts. AJR Am J Roentgenol 211:W267–W274 30240292 10.2214/AJR.17.19355

[42] Jochelson MS, Pinker K, Dershaw DD et al (2017) Comparison of screening CEDM and MRI for women at increased risk for breast cancer: A pilot study. Eur J Radiol 97:37–43 29153365 10.1016/j.ejrad.2017.10.001

[43] Coffey K, Jochelson MS (2022) Contrast-enhanced mammography in breast cancer screening. Eur J Radiol 156:110513 36108478 10.1016/j.ejrad.2022.110513 PMC10680079

[44] Lehman CD, Arao RF, Sprague BL et al (2017) National performance benchmarks for modern screening digital mammography: update from the breast cancer surveillance consortium. Radiology 283:49–58 27918707 10.1148/radiol.2016161174 PMC5375631

[45] Hubbard RA, Kerlikowske K, Flowers CI, Yankaskas BC, Zhu W, Miglioretti DL (2011) Cumulative probability of false-positive recall or biopsy recommendation after 10 years of screening mammography: a cohort study. Ann Intern Med 155:481–492 22007042 10.1059/0003-4819-155-8-201110180-00004 PMC3209800

[46] Perry N, Broeders M, de Wolf C, Törnberg S, Holland R, von Karsa L (2008) European guidelines for quality assurance in breast cancer screening and diagnosis.: fourth edition -: summary document. Ann Oncol 19:614–622 18024988 10.1093/annonc/mdm481

[47] Hofvind S, Bennett RL, Brisson J et al (2016) Audit feedback on reading performance of screening mammograms: an international comparison. J Med Screen 23:150–159 26892191 10.1177/0969141315610790

[48] Dabbous FM, Dolecek TA, Berbaum ML et al (2017) Impact of a false-positive screening mammogram on subsequent screening behavior and stage at breast cancer diagnosis. Cancer Epidemiol Biomarkers Prev 26:397–403 28183826 10.1158/1055-9965.EPI-16-0524 PMC5336525

[49] Francis A, Thomas J, Fallowfield L et al (2015) Addressing overtreatment of screen detected DCIS; the LORIS trial. Eur J Cancer 51:2296–2303 26296293 10.1016/j.ejca.2015.07.017

[50] Elshof LE, Tryfonidis K, Slaets L et al (2015) Feasibility of a prospective, randomised, open-label, international multicentre, phase III, non-inferiority trial to assess the safety of active surveillance for low risk ductal carcinoma in situ - the LORD study. Eur J Cancer 51:1497–1510 26025767 10.1016/j.ejca.2015.05.008

[51] COMET. Available at: https://clinicaltrials.gov/ct2/show/NCT02926911. Accessed 13 September 2023.

[52] Muhlberger N, Sroczynski G, Gogollari A et al (2021) Cost effectiveness of breast cancer screening and prevention: a systematic review with a focus on risk-adapted strategies. Eur J Health Econ 22:1311–1344 34342797 10.1007/s10198-021-01338-5

[53] Khan SA, Hernandez-Villafuerte KV, Muchadeyi MT, Schlander M (2021) Cost-effectiveness of risk-based breast cancer screening: a systematic review. Int J Cancer. 10.1002/ijc.33593 10.1002/ijc.33593 33844853

[54] Newman LA (2022) Breast cancer screening in low and middle-income countries. Best Pract Res Clin Obstet Gynaecol 83:15–23 35589536 10.1016/j.bpobgyn.2022.03.018

[55] Shah BA, Mirchandani A, Abrol S (2022) Impact of same day screening mammogram results on women’s satisfaction and overall breast cancer screening experience: a quality improvement survey analysis. BMC Womens Health 22:338 10.1186/s12905-022-01919-3 PMC9361536 35941606

[56] Mathioudakis AG, Janner J, Moberg M, Alonso-Coello P, Vestbo J (2019) A systematic evaluation of the diagnostic criteria for COPD and exacerbations used in randomised controlled trials on the management of COPD exacerbations. ERJ Open Res 5:00136–2019 31754621 10.1183/23120541.00136-2019 PMC6856493

[57] Rainey L, van der Waal D, Broeders MJM (2020) Dutch women’s intended participation in a risk-based breast cancer screening and prevention programme: a survey study identifying preferences, facilitators and barriers. BMC Cancer 20:965 33023516 10.1186/s12885-020-07464-2 PMC7539478

